# Expanding the genetic code of *Salmonella* with non-canonical amino acids

**DOI:** 10.1038/srep39920

**Published:** 2016-12-23

**Authors:** Qinglei Gan, Brent P. Lehman, Thomas A. Bobik, Chenguang Fan

**Affiliations:** 1 Department of Chemistry and Biochemistry, University of Arkansas, Fayetteville, AR, 72701, USA; 2 Roy J. Carver Department of Biochemistry, Biophysics and Molecular Biology, Iowa State University, Ames, IA, 50011, USA

## Abstract

The diversity of non-canonical amino acids (ncAAs) endows proteins with new features for a variety of biological studies and biotechnological applications. The genetic code expansion strategy, which co-translationally incorporates ncAAs into specific sites of target proteins, has been applied in many organisms. However, there have been only few studies on pathogens using genetic code expansion. Here, we introduce this technique into the human pathogen *Salmonella* by incorporating *p*-azido-phenylalanine, benzoyl-phenylalanine, acetyl-lysine, and phosphoserine into selected *Salmonella* proteins including a microcompartment shell protein (PduA), a type III secretion effector protein (SteA), and a metabolic enzyme (malate dehydrogenase), and demonstrate practical applications of genetic code expansion in protein labeling, photocrosslinking, and post-translational modification studies in *Salmonella*. This work will provide powerful tools for a wide range of studies on *Salmonella*.

Non-canonical amino acids (ncAAs) are powerful tools for protein studies. In the past few years, more than 150 different ncAAs have been incorporated into proteins in both prokaryotic and eukaryotic organisms using varied approaches. One of the most powerful methods of ncAA incorporation is genetic code expansion^1^,^2^,^3^,^4^,^5^,^6^,^7^,^8^. This approach uses an orthogonal aminoacyl-tRNA synthetase (AARS)/tRNA pair, which does not cross-react with host AARSs and tRNAs, to direct the incorporation of an ncAA at an assigned codon (usually the amber stop codon). This allows the introduction of ncAAs with novel functional groups at a precise position in a protein of interest. Scientists have already used this strategy to insert photocrosslinkers for mapping weak, transient, or pH sensitive protein interactions, to install post-translational modifications for regulating biological processes or identifying modifying enzymes, to incorporate photo-caged amino acids for controlling signaling and channeling by light, and to introduce biophysical probes and labels for providing exquisite insights into protein dynamics. These approaches have been combined with imaging systems, single-molecule studies, biophysical techniques, structural biology, and mass spectrometry to answer biological questions that are difficult or impossible to address by most classical methods^9^,^10^,^11^,^12^,^13^,^14^.

As the key components of genetic code expansion, a number of orthogonal AARS/tRNA pairs have been developed. In *Escherichia coli*, three pairs have been most successful: (1) an evolved orthogonal pair based on the *Methanocaldococcus jannaschii* tyrosyl-tRNA synthetase (*mj*TyrRS) and its cognate tRNA, which has been used to install a diverse array of tyrosine and phenylalanine derivatives^15^; (2) a natural orthogonal pair of pyrrolysyl-tRNA synthetase (PylRS) and its cognate tRNA from *Methanosarcinaceae* species, which facilitates the incorporation of a variety of lysine and phenylalanine analogs^16^,^17^; and (3) the phosphoseryl-tRNA synthetase (SepRS) and its cognate tRNA from methanogenic archaea, which were engineered for phosphoserine incorporation^18^,^19^.

*Salmonella* infects both human and animals, causing millions of illnesses every year. It is also an important model organism for gastrointestinal and extraintestinal pathogenesis studies. Due to its significance in public health, we sought to expand the genetic code in *Salmonella* with a variety of ncAAs, increasing the diversity of approaches that can be used in *Salmonella* studies. We utilized the *mj*TyrRS, PylRS, and SepRS systems to incorporate *p*-azido-phenylalanine, benzoyl-phenylalanine, acetyl-lysine, and phosphoserine in *Salmonella*, individually. For validation, we incorporated these ncAAs into *Salmonella* native proteins including a microcompartment shell protein, a type III secretion effector protein, and a TCA cycle enzyme respectively to demonstrate practical applications of genetic code expansion in protein labeling, photocrosslinking, and post-translational modification studies in *Salmonella*.

## Results

### Expression of target proteins and efficiency of orthogonal pairs in *Salmonella*

**Figure 1:**
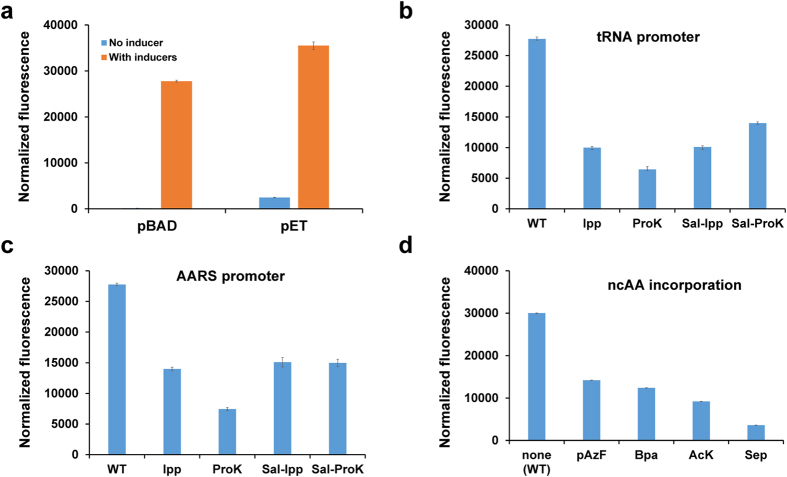
Expression of target proteins and noncanonical amino acid incorporation efficiency in *Salmonella*. (**a**) Comparison of the Para and Plac promoters to determine optimal superfolder green fluorescent protein (sfGFP) expression; (**b**) Comparison of the promoters to determine optimal tRNA expression for p-azido-phenylalanine (pAzF) incorporation: WT represents full-length wild-type sfGFP; (**c**) Comparison of the promoters to determine optimal aminoacyl-tRNA synthetase expression for pAzF incorporation: WT represents full-length wild-type sfGFP; (**d**) Comparison of the incorporation of noncanonical amino acids (ncAAs) at the position 151 of sfGFP: None (WT) represents full-length wild-type sfGFP without any ncAA supplied in the growth media. The normalized fluorescence was the absolute fluorescence readings at 12 h normalized by the corresponding cell densities. The mean values and standard deviations were calculated from three replicates.

First, we compared the expression of target proteins from different vectors in *Salmonella*. We used superfolder green fluorescent protein (sfGFP) as a reporter, which was engineered to have improved tolerance of circular permutation, greater resistance to chemical denaturants, and enhanced folding kinetics^20^, and put its gene in either a modified pBAD vector with the arabinose promoter or a modified pET vector with the *lac* promoter. For using the arabinose promoter, we removed the *araBAD* gene cluster which encodes arabinose metabolic enzymes from the genome. Although the pET vector had higher expression of sfGFP, the background of the pBAD vector was much lower (Fig. 1a). Furthermore, the expression of sfGFP in the pBAD vector can be modulated by the concentration of arabinose in the media (Supplementary Fig. S1). So we chose the pBAD vector to express target proteins in later experiments.

To allow varied applications, we chose four orthogonal pairs of AARS/tRNA that are widely used in *E. coli*: (1) a recently evolved *mj*TyrRS variant which is highly specific for *p*-azido-phenylalanine (pAzF) incorporation^21^; (2) an optimized *mj*TyrRS variant for incorporating a photocrosslinker, benzoyl-phenylalanine (Bpa); (3) an optimized PylRS system for the introduction of an important post-translational modification, acetyl-lysine (AcK)^22^; and 4) an optimized SepRS system for the incorporation of phosphoserine (Sep), which is another major post-translational modification^23^.

There are considerable genetic differences between *E. coli* and *Salmonella* which may affect the incorporation efficiency of ncAAs in *Salmonella* by these orthogonal pairs which were developed in *E. coli*. To modify these pairs for use in *Salmonella*, we first optimized their expression. In *E. coli*, the genes of orthogonal AARSs and tRNAs are constructed with the *lpp* promoter before AARS genes and the *ProK* promoter before tRNA genes^22^. Sequence alignments showed that the *lpp* promoter in *Salmonella* is highly similar to that in *E. coli,* but the *ProK* promoters are quite different between these two organisms (Supplementary Fig. S2). Thus, we compared the effect of tRNA promoters on the incorporation of pAzF by using a sfGFP-based fluorescence assay^24^. By site-directed mutagenesis, a stop codon (UAG) was introduced at position 151 of the sfGFP gene which was shown to be permissive at that site^22^,^24^,^25^. Higher pAzF incorporation results in better readthrough of the stop codon thus generating higher fluorescence. The results showed that the *Salmonella ProK* promoter (*Sal-ProK*) gave the highest ncAA incorporation (Fig. 1b). A similar approach was applied to test the effect of AARS promoters, and the results showed the *Salmonella lpp* promoter (*Sal-lpp*) had the best efficiency (Fig. 1c). To further optimize AARS and tRNA expression based on varying promoter strength, we made a matrix of 16 combinations of four promoters for tRNA expression and four promoters for AARS expression. The results also showed that the combination of *Sal-lpp* for AARS expression and *Sal-ProK* for tRNA expression was the best pair (Supplementary Table S1). Thus, we put the AARS genes after *Sal-lpp* promoter and the tRNA genes after the *Sal-ProK* promoter. With the same fluorescence assay, we tested the incorporation of the other three ncAAs mentioned above. Promisingly, the incorporation efficiency of pAzF, Bpa, and AcK was about 30% in average compared to the expression of the wild-type sfGFP (Fig. 1d). The incorporation of Sep was relatively low at ~15%, similar to that in *E. coli*, but it could still produce ~50 mg protein in 1 L media which was sufficient for further studies. Finally, all the ncAA incorporation was confirmed with mass spectrometry (MS) analyses (Supplementary Fig. S3).

### Incorporation of *p*-azido-phenylalanine (pAzF) as a handle for protein labeling by click chemistry

Currently, the most popular tags to label proteins for imaging purposes are fluorescent proteins (FPs)^26^. However, there are also disadvantages of this method. First, the relatively large size of FPs may perturb the structures and functions of target proteins. Second, FP fusions are limited to the N- or C- termini of target proteins. Moreover, FPs need oxygen to produce fluorescence. Here, we applied a protein labeling method based on the incorporation of pAzF, which is then reacted with an alkyne-fluorescent dye by the click reaction to produce fluorescence without oxygen. Moreover, due to its small size, pAzF can be incorporated at essentially any site within a protein by using a UAG codon and the optimized pAzF incorporation system^21^.

**Figure 2:**
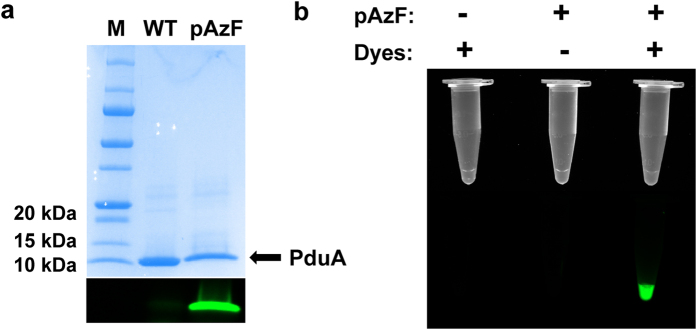
Incorporation of p-azido-phenylalanine (pAzF) in *Salmonella* PduA protein. (**a**) The SDS-PAGE gel of PduA labeling. The upper panel is the gel stained with Coomassie Blue. The lower panel was captured with fluorescent filters for Alexa Fluor 488. (**b**) The fluorescence labeling of purified Pdu microcompartments *in vitro*. The upper panel was captured without filters. The lower panel was captured with fluorescent filters for Alexa Fluor 488. The left lane was wild-type Pdu MCPs with dyes. The middle lane only contained pAzF-containing Pdu MCPs. The right lane included both pAzF-containing Pdu MCPs and dyes.

As for the target protein for validation, we chose a native *Salmonella* protein, PduA, one of the major shell proteins of 1,2-propanediol utilization (Pdu) microcompartments (MCPs) which are large multi-protein complexes, functioning as organelles for 1,2-propanediol degradation^27^. Based on the crystal structure of PduA^28^, residue N67 which is at the outer surface of Pdu MCPs was selected for pAzF incorporation. First, we inserted an amber stop codon (UAG) at the position 67 of PduA and overexpressed PduA from the pBAD plasmid. The purified pAzF-containing PduA was labeled by a fluorescent dye with an alkyne group through click chemistry^29^. With a 5-minute reaction, the pAzF-containing PduA protein gave a clear and bright green band, while the wild-type PduA protein had a very low background (Fig. 2a). Then, we used the same approach to label Pdu MCPs in living cells of *Salmonella*. We mutated the position 67 of PduA gene to an amber stop codon in the genome, expressed the entire *pdu* operon to produce pAzF-containing Pdu MCPs, which were then purified by previous protocols^30^, and labeled with fluorescent dyes. After washing away excess fluorescent dyes, only pAzF-containing MCPs had fluorescence (Fig. 2b). The SDS-PAGE gel also indicated the specific labeling of PduA in the whole Pdu MCP protein profile (Supplementary Fig. S4). The pAzF incorporation in PduA was confirmed by LC-MS/MS analysis (Supplementary Fig. S5).

### Incorporation of benzoyl-phenylalanine (Bpa) for photocrosslinking

Understanding how proteins interact is one of the most common questions to be solved for any biological processes. However, it can be difficult when the interactions are weak, transient, pH-dependent, or when the interactions are at particular subcellular locations such as membranes. The covalent nature of photocrosslinking enables detection of low-affinity interactions. Moreover, it can be used in living cells to identify specific, direct protein-protein interactions^31^. Benzoyl-l-phenylalanine (Bpa) is a widely used photocrosslinker excited by UV at the wavelength of 365 nm. Here, we utilized an optimized *mj*TyrRS variant^32^,^33^ to incorporate Bpa into proteins in *Salmonella*.

**Figure 3:**
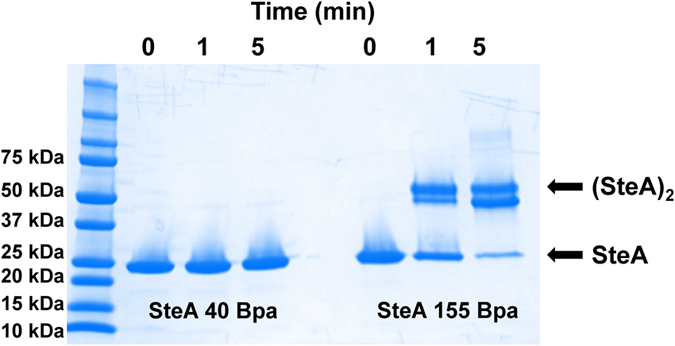
Incorporation of benzoyl-phenylalanine (Bpa) in *Salmonella* SteA protein. The SAS-PAGE gel for photocrosslinking of SteA proteins with Bpa incorporated at position 40 and 155, individually. The 365 nm UV irritation time was 0, 1 and 5 minutes. With 1-minute exposure, about 60% of total proteins were cross-linked, while 5-minute exposure made more than 95% of total protein cross-linked. The expected SteA monomer and SteA dimer were indicated at their corresponding molecular weights.

As for the target protein for validation, we chose a native *Salmonella* protein, SteA, a type III secretion effector which contributes to the control of membrane dynamics of *Salmonella*-containing vacuoles^34^, globally affecting host cell proliferation, morphology, adhesion and migration^35^. For minimal perturbation to the protein structure, we selected two tyrosine residues at positions 40 and 155 for Bpa incorporation, individually. First, we mutated these two positions to the amber stop codon separately, and overexpressed SteA from the pBAD plasmid. Then, we exposed the purified Bpa-containing SteA proteins to the long wavelength 365 nm UV light and analyzed the products by SDS-PAGE. Interestingly, the SteA (155-Bpa) formed a covalent dimer, while the SteA (40-Bpa) showed no crosslinking (Fig. 3). This indicated that residue Tyr155 may be at the dimer interface, so the SteA (155-Bpa) variant may affect further experiments to identify SteA-interacting proteins *in vivo*, while SteA (40-Bpa) variant should be ideal for this purpose. The Bpa incorporation in SteA was confirmed by LC-MS/MS analyses (Supplementary Fig. S5).

### Incorporation of acetyl-lysine (AcK) and phosphoserine (Sep) for studying protein post-translational modifications

Acetylation and phosphorylation are two of the most common post-translational modifications in natue^36^,^37^. Previous studies have shown that lysine acetylation regulates the functions of many proteins in *Salmonella*^38^,^39^,^40^. Although proteomic analyses revealed that more than 20% of proteins in bacteria are modified by acetylation or phosphorylation^41^,^42^, limited validation has been done to confirm these modifications. One of the challenges is that it is difficult to synthesize proteins that are fully modified at desired positions by most classical methods. Therefore, we applied the genetic code expansion strategy to directly incorporate AcK and Sep site-specifically into proteins in *Salmonella* to overcome such challenge.

**Figure 4:**
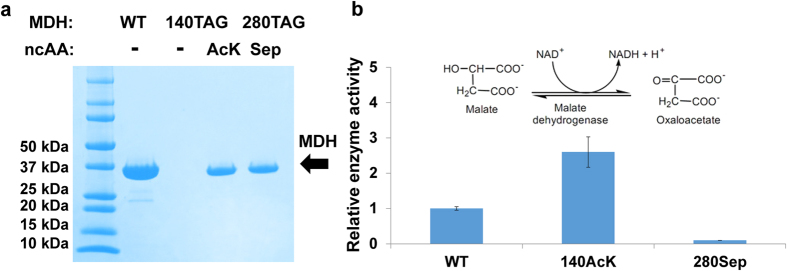
Incorporation of acetyl-lysine (AcK) and phosphoserine (Sep) in *Salmonella* malate dehydrogenase (MDH). (**a**) The SDS-PAGE gel of purified MDH and its modified variants. From left to right, lane 1 is the protein marker; lane 2 is wild-type full-length MDH; lane 3 is MDH with 140 position mutated to a UAG stop codon and without any noncanonical amino acid supplied in the growth media; lane 4 is MDH with 140 position mutated to a UAG stop codon and with 5 mM AcK supplied in the growth media; lane 5 is MDH with 280 position mutated to a UAG stop codon and with 2 mM Sep supplied in the growth media. (**b**) The enzyme activities of the MDH enzyme and its modified variants. The mean values and standard deviations were calculated from three replicates. The enzyme activity of wild-type MDH was set as 1, and the reaction catalyzed by MDH is demonstrated as inset.

As for the target protein for validation, we chose a native *Salmonella* protein, malate dehydrogenase (MDH), an important metabolic enzyme in the TCA cycle^43^. Previous studies have shown that the acetylation of lysine residues in mammalian MDHs increases their enzyme activities, and is involved in the cross-talk between adipogenesis and the intracellular energy level^44^,^45^. It has also been shown that bacterial MDHs are subject to both lysine acetylation and serine phosphorylation^38^,^46^. We selected residue Lys140 for AcK incorporation based on the proteomic studies^38^, and residue Ser280 for Sep incorporation, as its corresponding position in *E. coli* MDH (95% sequence identity) was shown to be phosphorylated^46^. We mutated these two positions to amber stop codons separately, and overexpressed the MDH from pBAD plasmids (Fig. 4a). Enzyme assays of the purified MDH proteins showed that acetylation of lysine140 increased enzyme activity, while the phosphorylation of Ser280 inactivated the enzyme (Fig. 4b), indicating that *Salmonella* cells could use different modifications to activate or inactivate the MDH enzyme to control the flux in the TAC cycle. The AcK and Sep incorporation in MDH was confirmed by LC-MS/MS analyses (Supplementary Fig. S5).

## Discussion

In this work, we used *mj*TyrRS to incorporate pAzF and Bpa into two different native *Salmonella* proteins. In other organisms, the *mj*TyrRS has been engineered to incorporate many other tyrosine or phenylalanine analogs^2^ that enable a range of biochemical investigations^1^,^2^,^9^,^10^,^14^: (1) Acetyl-phenylalanine (ketone group), allyl-tyrosine (alkene group), and propargyloxy-phenylalanine (alkyne group) can be modified by selective chemical reactions such as oxime condensation and click chemistry to allow site-specific protein labeling; (2) Cyano-phenylalanine, amino-phenylalanine, iodo-phenylalanine, and fluoro-phenylalanine can be used as heavy atoms for X-ray structure determination and probes for IR and NMR; (3) Photo-reactive amino acids such as bipyridyl-alanine, benzoyl-phenylalanine, and nitrobenzyl-tyrosine can be used as switches to control biological processes with light; (4) Varied post-translation modifications such nitro-tyrosine and sulfo-tyrosine can be used to study functions of protein modifications. Since we have successfully introduced the *mj*TyrRS systems for pAzF and Bpa incorporation in this study, the ncAAs mentioned above might also be utilized in *Salmonella* for a wide range of biochemical and biophysical investigations.

We also used the PylRS system to install lysine acetylation into the MDH protein of *Salmonella*. This system has been widely used in both prokaryotic and eukaryotic organisms, and has been evolved for not only lysine derivatives but also phenylalanine or even tryptophan analogs^16^,^17^. Other lysine modifications such as methylation and ubiquitylation have also be incorporated or generated by the PylRS systems to form complete lysine modification profiles^47^,^48^. A previous study also showed that the PylRS system can incorporate pyrrolysine analogs as crosslinkers in *Salmonella*^49^. Thus, the *mj*TyrRS and PylRS systems, open up a wide range of ncAA candidates with different sizes and properties for a variety of research objectives.

Proteomic studies indicate many proteins have both acetylation and phosphorylation modifications simultaneously. We have demonstrated the facile incorporation of lysine acetylation and serine phosphorylation site-specifically into proteins, individually. Moreover, PylRS and its variants have low selectivity toward the tRNA anticodon, and can be used for incorporating ncAAs towards different codons such as the opal stop codon (TGA), quadruple codons, and even sense codons^17^. Thus, combining two mutually orthogonal PylRS and SepRS systems with different stop codons or quadruplet codons, could allow the production of simultaneously acetylated and phosphorylated proteins, providing a powerful tool to study the crosstalk between protein acetylation and phosphorylation.

Our established ncAA incorporation systems could facilitate a number of studies on *Salmonella*, such as tracking proteins, mapping protein-protein interaction networks, and characterizing protein post-translational modifications. Together with this work, genetic code expansion has been successfully applied in several pathogens including *Salmonella, Shigella*, and *Mycobacterium*^49^,^50^, suggesting that it is promising to extend this strategy to other pathogens for broader applications. Furthermore, all the ncAAs and chemicals mentioned in this work are commercially available, so our systems could benefit many biological laboratories without the need for in house organic synthesis.

## Methods

### General molecular biology

The amino acids in this study were purchased from Sigma-Aldrich or ChemImpex. *E. coli* TOP10 cells (Life Technologies) were used for general cloning. *Salmonella enterica* Serovar Typhimurium LT2 was used as the representative *Salmonella* strain. Plasmids: The genes of AARSs and tRNAs were cloned by PCR from laboratory inventory and inserted into the pTech plasmid. The genes of target proteins with C-terminal His_6_-tag were cloned into the pBAD plasmid with the arabinose promoter, or a modified pET plasmid with the *lac* promoter. All the cloning experiments were performed by the Gibson Assembly kit (New England Biolabs). The mutations of target genes were made by the QuikChange II mutagenesis kit (Agilent Life Sciences). The SDS-PAGE gel is 4–20% gradient gel purchased from Bio-Rad. The chromosomal mutation PduA N67TAG was constructed as described previously^51^. DNA oligos used to construct the mutation were ordered from Integrated DNA Technologies (Coralville, IA). The mutation was confirmed by DNA sequencing. The information of plasmids and primers was listed in Supplementary Tables S2 and S3, respectively. The primary sequences of proteins were listed in the Supplementary information, and the locations of the ncAA substitution and the active sites of enzymes were marked as well.

### sfGFP readthrough assay

The strains harboring the genes of sfGFP as well as AARSs and tRNAs were inoculated into 2 ml LB medium. The overnight culture was diluted with fresh LB medium to an absorbance of 0.2 at 600 nm, supplemented with ncAAs. 1 mM arabinose or 1 mM IPTG was added to induce the expression of sfGFP. 200 μL culture of each strain was transferred to a well in the 96 well plate. Cells were shaken for 24 hours at 37 °C, with monitoring of fluorescence intensity (excitation 485 nm, emission 528 nm, bandwidths 20 nm) and optical cell density by the microplate-reader.

### Protein expression and purification

The genes of target proteins were cloned into the pBAD vector with a C-terminal His_6_-tag, and transformed into LT2 *ΔaraBAD* cells together with the plasmids harboring genes of AARSs and tRNAs for expression. The expression strain was grown on 1 L of LB medium at 37 °C to an absorbance of 0.6–0.8 at 600 nm, and protein expression was induced by the addition of 1 mM arabinose and ncAAs (1 mM for pAzF, Bpa; 2 mM for Sep; and 5 mM for AcK). Cells were incubated at 30 °C for an additional 8 hours, and harvested by centrifugation. The cell paste was suspended in 15 ml of 50 mM Tris (pH 7.5), 300 mM NaCl, 20 mM imidazole, and broken by sonication. The crude extract was centrifuged at 20,000 × g for 30 min at 4 °C. The soluble fraction was loaded onto a column containing 2 ml of Ni-NTA resin (Qiagen). The column was then washed with 50 ml of 50 mM Tris (pH 7.5), 300 mM NaCl, 50 mM imidazole, and eluted with 2 ml of 50 mM Tris (pH 7.5), 300 mM NaCl, 200 mM imidazole.

### Mass spectroscopy analysis

The samples was loaded onto the SDS-PAGE gel. The bands with the corresponding molecular weight of target proteins were cut and sent for MS analysis. The proteins were trypsin digested by a standard in-gel digestion protocol, and analyzed by LC-MS/MS on an LTQ Orbitrap XL (Thermo Scientific) equipped with a nanoACQUITY UPLC system (Waters). A Symmetry C18 trap column (180 μm × 20 mm; Waters) and a nanoACQUITY UPLC column (1.7 μm, 100 μm × 250 mm, 35 °C) were used for peptide separation. Trapping was done at 15 μL min^−1^, 99% buffer A (0.1% formic acid) for 1 min. Peptide separation was performed at 300 nL min^−1^ with buffer A and buffer B (CH_3_CN containing 0.1% formic acid). The linear gradient was from 5% to 50% buffer B at 50 min, to 85% buffer B at 51 min. MS data were acquired in the Orbitrap with one microscan, and a maximum inject time of 900 ms followed by data-dependent MS/MS acquisitions in the ion trap (through collision induced dissociation, CID). The Mascot search algorithm was used to determine the amino acid composition at specific positions (Matrix Science, Boston, MA).

### Protein labeling

The fluorescent labeling reagent was Click-IT^®^ Alexa Fluor^®^ 488 DIBO Alkyne purchased from Invitrogen. The labeling experiment was performed by following the protocol from the manufacturer. 100 μg purified PduA protein or 1 mg purified *Salmonella* microcompartment was used for labeling. The labeled proteins were separated by the SDS-PAGE gel and imaged by the ChemiDoc^TM^ MP System from Bio-Rad.

### Photocrosslinking

The reactions were performed in a 96-well plate by using 100 μl of proteins with the concentration of 1 mg/ml. Samples were irradiated at 365 nm by using the UV crosslinker CL-1000L (Denville Scientific). Then, samples were removed from the wells and analyzed by the SDS-PAGE gel.

### MDH activity assay

The assays were performed by following the instruction of the EnzyChrom^TM^ Malate Dehydrogenase Assay Kit (EMDH-100) from BioAssay Systems. This non-radioactive, colorimetric assay is based on the reduction of the tetrazolium salt MTT in a NADH-coupled enzymatic reaction to a reduced form of MTT which exhibits an absorption maximum at 565 nm. The increase in absorbance at 565 nm is proportional to the enzyme activity. 100 μg purified MDH and its variants were used in the assay.

## Additional Information

**How to cite this article**: Gan, Q. *et al*. Expanding the genetic code of *Salmonella* with non-canonical amino acids. *Sci. Rep.* **6**, 39920; doi: 10.1038/srep39920 (2016).

**Publisher's note:** Springer Nature remains neutral with regard to jurisdictional claims in published maps and institutional affiliations.

## Supplementary Material

Supplementary Information
