# Supplementary material for: Expanding the genetic code of Salmonella with non-canonical amino acids
Source: Sci Rep. 2016 Dec 23;6:39920. doi: 10.1038/srep39920 (PMC5180212; doi:10.1038/srep39920)
Supplement: Supplementary Information [file srep39920-s1.pdf]

Expanding the genetic code of Salmonella with non-canonical amino acids

Qinglei Gan[1], Brent P. Lehman[2], Thomas A. Bobik[2] and Chenguang Fan[1,*]

Supplementary information

Protein sequences. The positions for noncanonical amino acid incorporation are marked in yellow. If the protein is an enzyme, active sites are underlined.

1) sfGFP
MSKGEELFTGVVPILVELDGDVNGHKFSVRGEGEGDATNGKLTLKFICTTGKLPVPWPTLVTTL
TYGVQCFSRYPDHMKRHDFFKSAMPEGYVQERTISFKDDGTYKTRAEVKFEGDTLVNRIELKG
IDFKEDGNILGHKLEYNFNSHNVYITADKQKNGIKANFKIRHNVEDGSVQLADHYQQNTPIGDG
PVLLPDNHYLSTQSVLSKDPNEKRDHMVLLEFVTAAGITHGMDELYKGS

2) PduA
MQQEALGMVETKGLTAAIEAADAMVKSANVMLVGYEKIGSGLVTVIVRGDVGAVKAATDAGAA
AARNVGEVKAVHVIPRPHTDVEKILPKGISQ

3) SteA
MPYTSVSTYARALSGNKLPHVAAGDYENKLSTKIMKGILYVLTAGLAYGFTRVIEHYCNVTPKVA
EFCANAGNIHNHLADAVRDGLFTIDVELSDGRMLTFEQLSLIAEGKPIVRISDGEHTVEVEGTFE
EICMRLEEGFFEAPAYYDYDIDEKYKTVRERMAAYNALPQALGAIPCLEYYIARASNMQEAKAQ
WAADIKARYHNYLDNY

4) MDH
MKVAVLGAAGGIGQALALLLKNQLPSGSELSLYDIAPVTPGVAVDLSHIPTAVKIKGFSGEDATP
ALEGADVVLISAGVARKPGMDRSDLFNVNAGIVKNLVQQIAKTCPKACVGIITNPVNTTVAIAAE
VLKKAGVYDKNKLFGVTTLDIIRSNTFVAELKGKLPTEVEVPVIGGHSGVTILPLLSQIPGVSFTE
QEAAELTKRIQNAGTEVVEAKAGGGSATLSMGQAAARFGLSLVRALQGEKGVVECAYVEGDG
QYARFFSQPLLLGKNGVEERKSIGTLSAFEQHSLDAMLDTLKKDIQLGEDFINK

Table S1. Comparison of fluorescence with the combinations of promoters for AARS and tRNA expression to determine optimal p-azido-phenylalanine incorporation in the position 151 of sfGFP. The normalized fluorescence was the absolute fluorescence readings at 12h normalized by the corresponding cell densities. The mean values and standard deviations were calculated from three replicates.

**AARS promoter**	**tRNA promoter**			
	Ipp	Sal-Ipp	ProK	**Sal-ProK**
Ipp	9995 ± 333	10096 ± 456	6435 ± 200	13980 ± 205
**Sal-Ipp**	9873 ± 109	10698 ± 420	6976 ± 749	**15096 ± 786**
ProK	6321 ± 575	6792 ± 298	5520 ± 341	7435 ± 260
Sal-ProK	10023 ± 475	9927 ± 650	7298 ± 176	14980 ± 609

Table S2. The list of plasmids used in this study.

	Antibiotic marker	Origin	Use	Source
**pBAD**	Ampicillin	colE1	Protein expression	Lab directory
**pET**	Ampicillin	colE1	Protein expression	Lab directory
**pTech**	Chloramphenicol	p15A	AARS/tRNA expression	Lab directory

Table S3. The list of primers used in this study.

Use	Primer sequences
**sfGFP 151TAG**	caattcgcacaacgtg**TAG**atcaccgcagataagc
**PduA 67TAG**	GCAGCCGCACGC**TAG**GTGGGTGAAGTG
**SteA 40TAG**	CATGAAAGGCATCTTG**TAG**GTGCTTACCGCAGGAC
**SteA 155TAG**	GATATTGATGAAAAA**TAG**AAAACCGTCAGAG
**MDH 140TAG**	GCCGGAGTCTACGAC**TAG**AACAAATTGTTTGGG
**MDH 280TAG**	GTTGAGGAACGTAAA**TAG**ATCGGCACCCTTAGC
**pET-Gibson-F**	CTCGAGGATCCGGCTGCTAACAAAGCCCGAAAGG
**pET-Gibson-R**	CATGGTATATCTCCTTCTTAAAGTTAAAC
**pBAD-Gibson-F**	CTCGAGATCTGCAGCTGGTACCATATGGGAATTCG
**pBAD-Gibson-R**	CCTAGGTTAATTCCTCCTGTTAGCCCAAAAAACGG
**sfGFP-Gibson-F**	GCTAACAGGAGGAATTAACCTAGGATGAGCAAGGGCGAAGAACTGTTTACGGGC
**sfGFP-Gibson-R**	CCATATGGTACCAGCTGCAGATCTCGAGTTAATGATGATGATGATGATG TGAGCCTTTATACAGTTCGTCCATACC
**PduA-Gibson-F**	GCTAACAGGAGGAATTAACCTAGGATGAGCAAACATCACCATCATCACCAC
**PduA-Gibson-R**	CCATATGGTACCAGCTGCAGATCTCGAGTTAATGATGATGATGATGATG TTATTGGCTAATACCCTTCGGTAAG
**SteA-Gibson-F**	GCTAACAGGAGGAATTAACCTAGGATGCCATATACATCAGTTTCTACCTATGCC
**SteA-Gibson-R**	CCATATGGTACCAGCTGCAGATCTCGAGTTAATGATGATGATGATGATG ATAATTGTCCAAATAGTTATGGTAGCGAGC
**MDH-Gibson-F**	GCTAACAGGAGGAATTAACCTAGGATGAAAGTGGCTGTGCTGGGTGCTGC
**MDH-Gibson-R**	CCATATGGTACCAGCTGCAGATCTCGAGTTAGTGATGGTGGTGATGATG CTTATTAATAAAATCCTCGCCAAGCTGGATG

Figure S1. The effect of arabinose concentrations on sfGFP expression in the pBAD plasmid. The normalized fluorescence was the absolute fluorescence readings at 12h normalized by the corresponding cell densities. The mean values and standard deviations were calculated from three replicates.

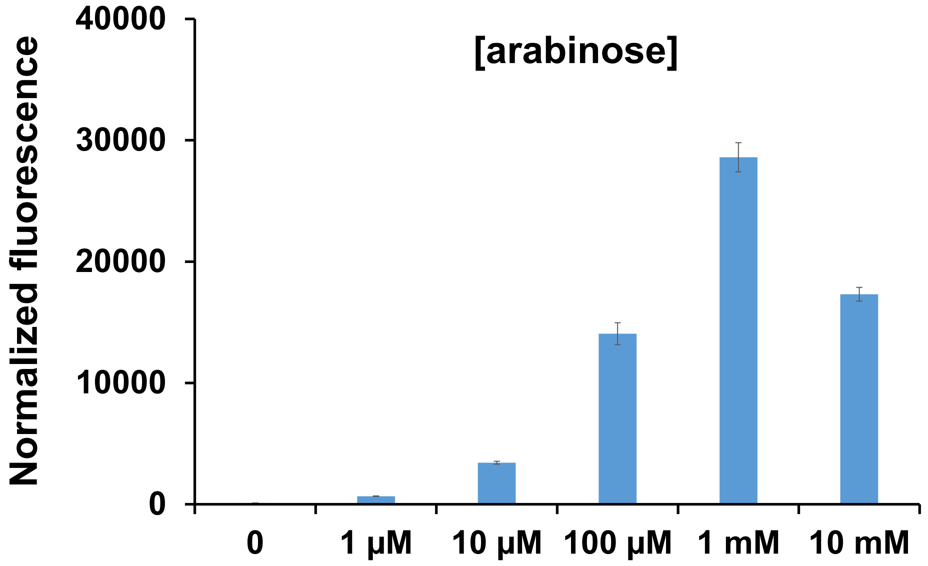

Figure S2. Alignment of *lpp* and *ProK* promoters and from *E. coli* K12 strain (Ec) and *Salmonella* LT2 strain (Sal). The differences were marked with red color.

```
Ec-lpp      CCCATCAAAAAAATATTCTCAACATAAAAAACTTTGTGTAATACTTGTAACGC
Sal-lpp     CCCATCAAAAAAATATTCTCAACATAAAAAAGTTTGTGTAATACTTGTAACGC

Ec-ProK     AGGCATTTTGCTATTAAGGGATTGACGAGGGCGTTC-TGCGCAGTAAGATGCGCCCCGCATT
Sal-ProK    TTGTATTTTGCGACTAAGGGATTGACGGGCGTGCGCCTGAGCAGTAAGATGCGCCC-GCATT
```

Figure S3. Mass spectrometry analysis of sfGFP protein containing noncanonical amino acids (ncAAs) expressed in *Salmonella*. Individual noncanonical amino acid incorporation was marked in figures. The amino acid sequence of the peptide is shown at the top of the figure, and the partial sequences of the peptides containing ncAAs can be read from the annotated b or y ion series. Amino acid Y^{TY} represents the p-azido-phenylalanine (pAzF). Amino acid Y^{TY} represents the benzoyl-phenylalanine (Bpa). Amino acid K^{AC} represents the acetyl-lysine (AcK). Amino acid S^{PH} represents the phosphoserine (Sep). The b ion series are the ions of peptides with the amino acid numbers counted from N-terminal of the peptide, while the numbers of the y ion series are counted from C-terminal of the peptide. The peptides of interest were marked with the m/z ratios.

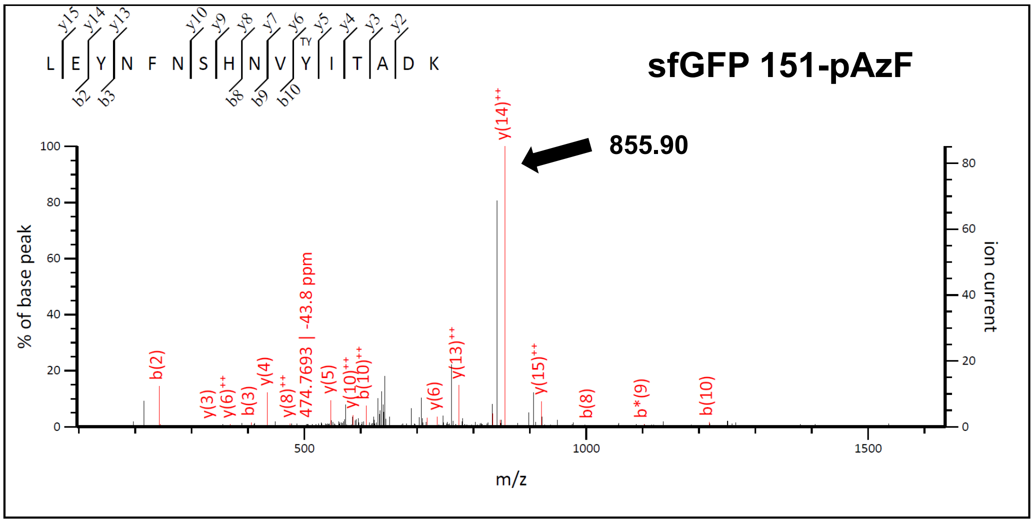

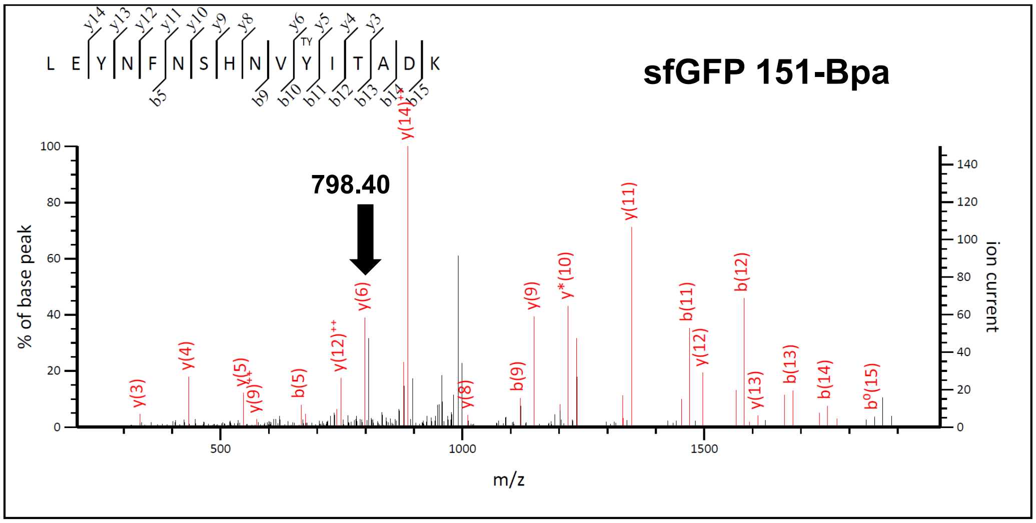

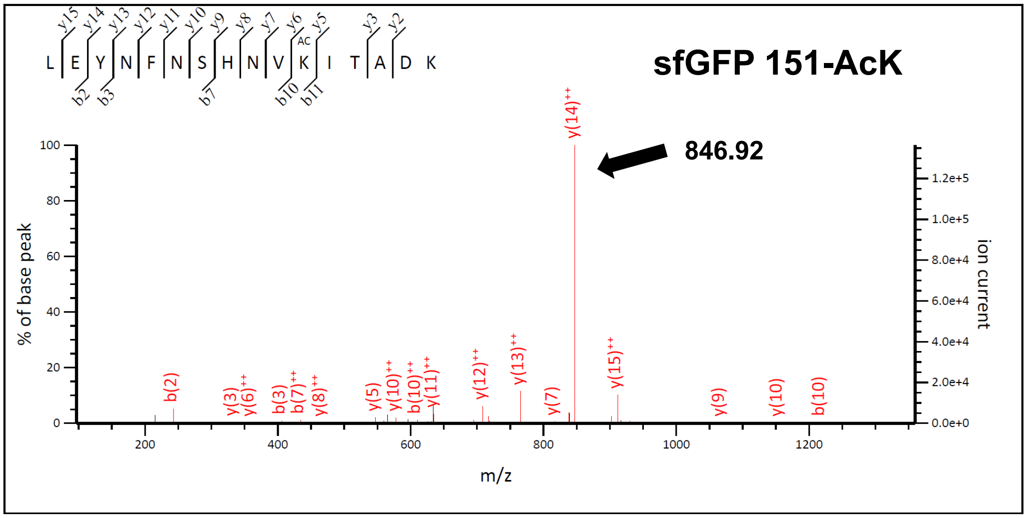

sfGFP 151-AcK
L E Y N F N S H N V K I T A D K
AC
846.92
% of base peak
ion current
m/z
y(14)++
y(15)++
y(13)++
y(12)++
b(2)

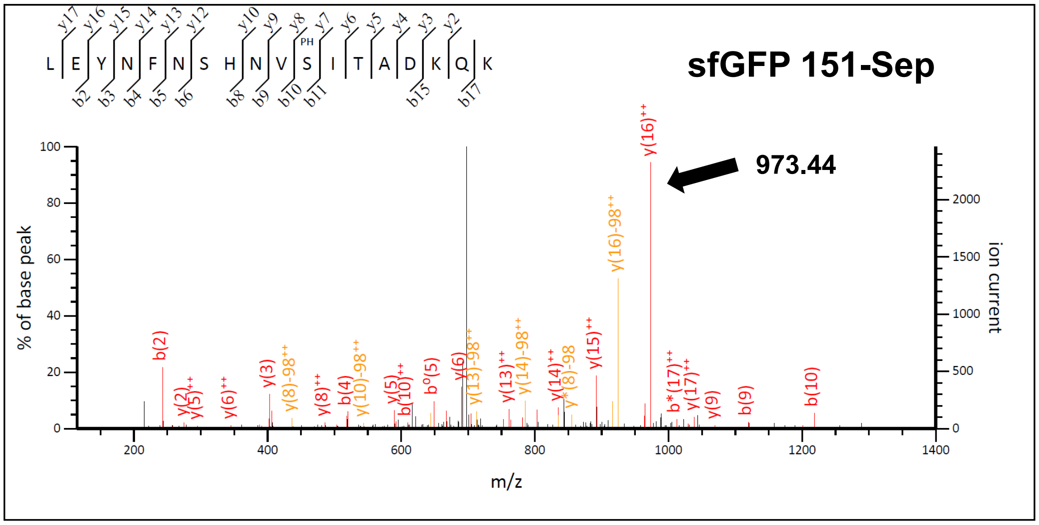

sfGFP 151-Sep
L E Y N F N S H N V S I T A D K Q K
PH
973.44
% of base peak
ion current
m/z
y(16)++
y(16)-98++
b(2)
y(15)++

Figure S4. The SDS-PAGE gel of labeled Pdu MCPs. The left panel is the gel stained with Coomassie Blue. The right panel was captured with fluorescent filters for Alexa Fluor 488.

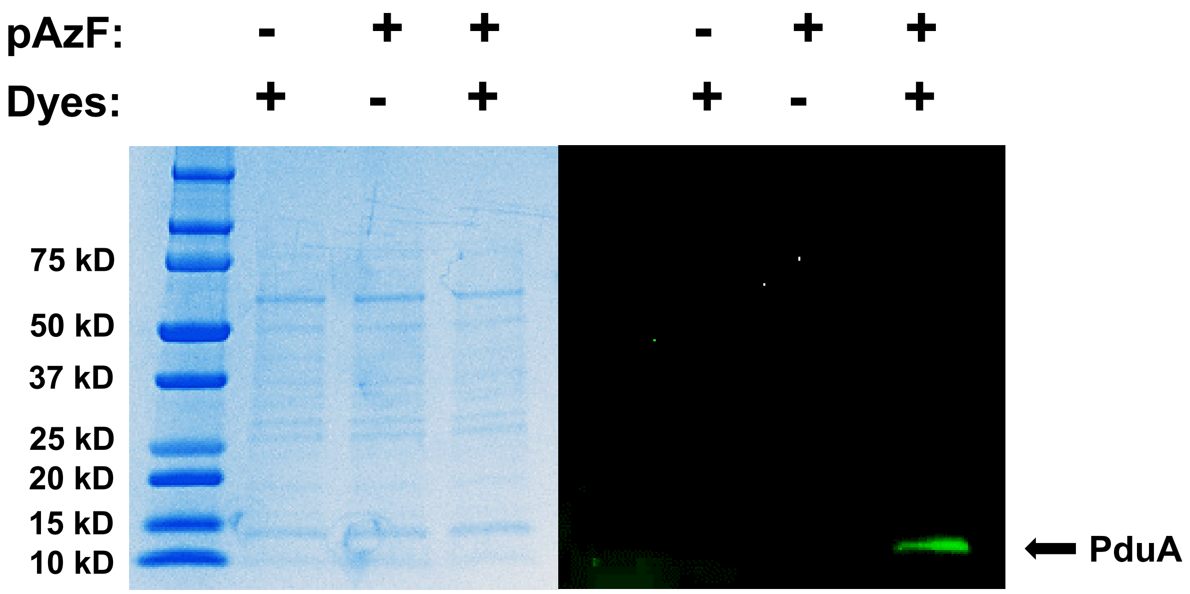

Figure S5. Mass spectrometry analysis of *Salmonella* native proteins containing noncanonical amino acids (ncAAs) expressed in *Salmonella*. Proteins with noncanonical amino acid incorporation were marked in figures. The amino acid sequence of the peptide is shown at the top of the figure, and the partial sequences of the peptides containing ncAAs can be read from the annotated b or y ion series. Amino acid N^{AS} represents the p-azido-phenylalanine (pAzF). Amino acid Y^{TY} represents the benzoyl-phenylalanine (Bpa). Amino acid K^{AC} represents the acetyl-lysine (AcK). Amino acid S^{PH} represents the phosphoserine (Sep). The b ion series are the ions of peptides with the amino acid numbers counted from N-terminal of the peptide, while the numbers of the y ion series are counted from C-terminal of the peptide. The peptides of interest were marked with the m/z ratios.

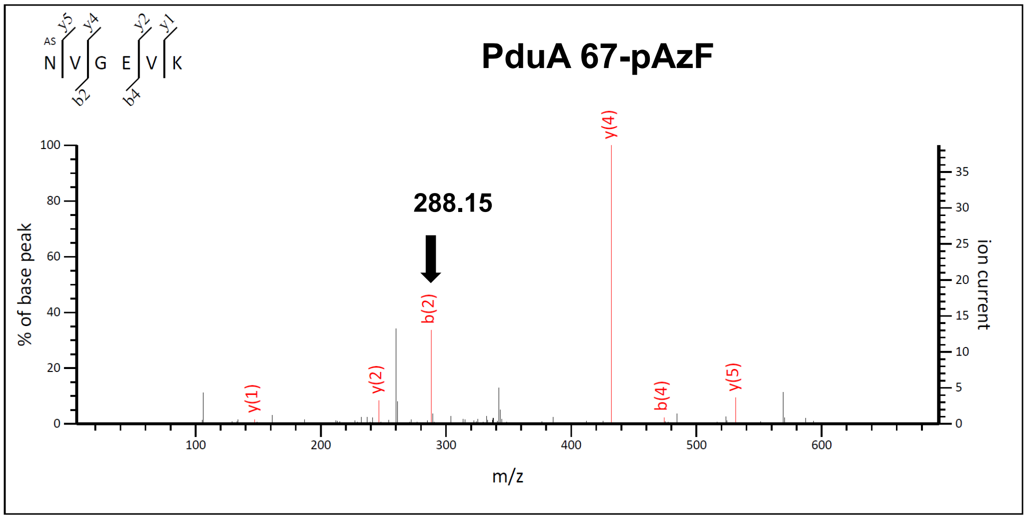

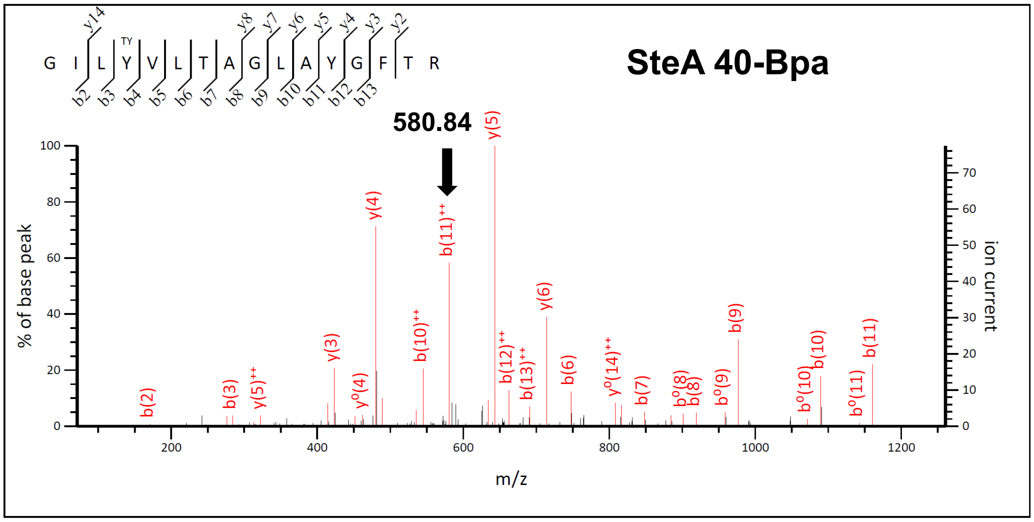
SteA 40-Bpa
G I L Y V L T A G L A Y G F T R
580.84
% of base peak
ion current
m/z

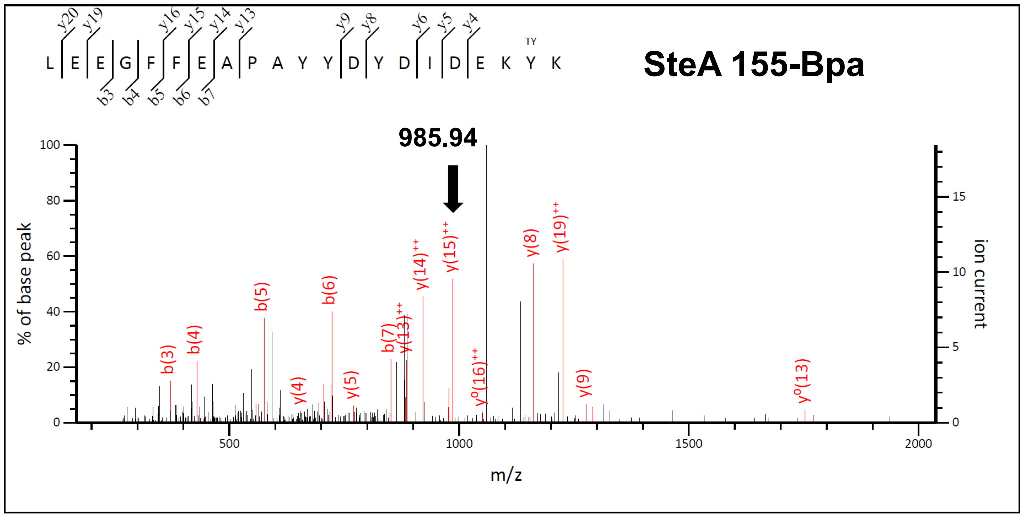
SteA 155-Bpa
L E E G F F E A P A Y Y D Y D I D E K Y K
985.94
% of base peak
ion current
m/z

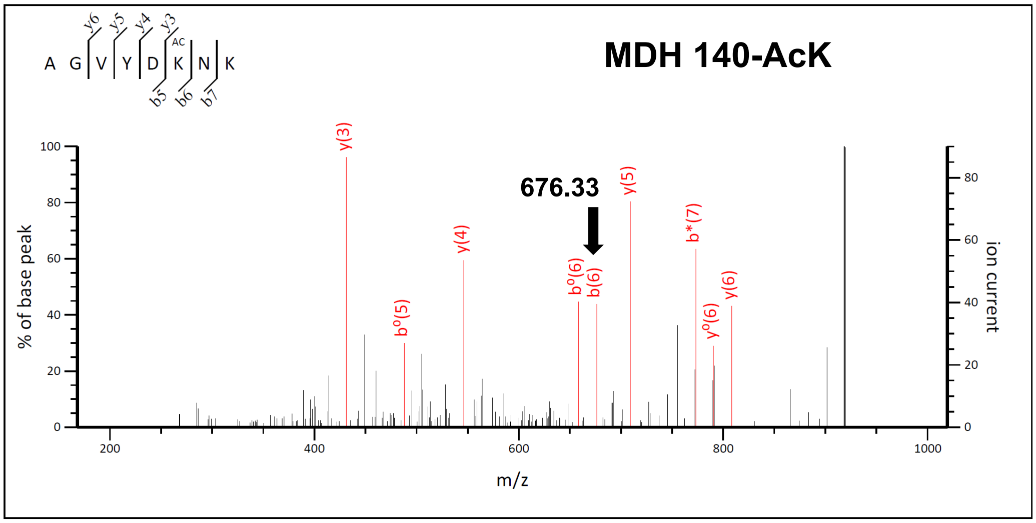

MDH 140-AcK
A G V Y D K N K
676.33
% of base peak
ion current
m/z

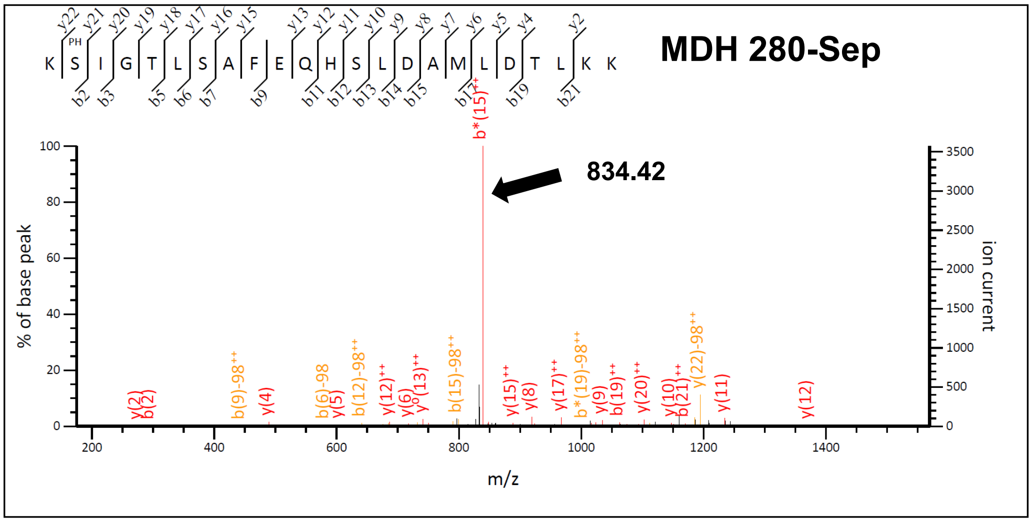

MDH 280-Sep
K S I G T L S A F E Q H S L D A M L D T L K K
834.42
% of base peak
ion current
m/z
